# Electrocardiogram abnormalities and associated factors among psychiatric patients attending follow up at Jimma Medical Center Psychiatry Clinic, Jimma, Ethiopia: an institution-based cross-sectional study

**DOI:** 10.1186/s12872-023-03092-3

**Published:** 2023-04-01

**Authors:** Betemariam Girma, Alemayehu Wondie, Wondwosen Debebe, Ahmed Juhar, Elsah Tegene, Deriba Bedane, Elias Mulat

**Affiliations:** 1 Department of Biomedical Sciences, College of Medicine and Health Sciences, Wolkite University, P.O. Box 07, Wolkite, Ethiopia; 2 Department of Biomedical Sciences, College of Medicine and Health Sciences, Wollo University, Dessie, Ethiopia; 3 Department of Internal Medicine, Institute of Health, Jimma University, Jimma, Ethiopia; 4 Department of Biomedical Sciences, Institute of Health, Jimma University, Jimma, Ethiopia

**Keywords:** Psychiatric disorders, Electrocardiogram, Psychotropic drugs, Ethiopia

## Abstract

**Background:**

Psychiatric patients have two to three-fold higher risk of cardiovascular morbidity and mortality as compared to the general population. Despite the high rate of cardiovascular disease, about 80% of patients with psychiatric disorders have fewer opportunities for cardiovascular disease screening. Early detection of subclinical cardiovascular disease using an electrocardiogram can improve the clinical outcomes of these patients. However, in Ethiopia, no previous study had been conducted on electrocardiogram abnormalities and associated factors among psychiatric patients. Hence, this study aimed to assess the electrocardiogram abnormalities and associated factors among psychiatric patients attending follow-up at Jimma Medical Center, Jimma, Ethiopia.

**Methods:**

An institution-based cross-sectional study was carried out among psychiatric patients attending Jimma Medical Center Psychiatry Clinic from October 14 to December 10, 2021. An interviewer-administered structured questionnaire was used to collect socio-demographic data, behavioral factors, disease-related and medication-related data. Anthropometry and blood pressure were measured following the standard protocols. A resting 12 lead ECG was recorded according to the standard recording protocol of the Minnesota code. Data were entered into Epi data version 4.6 and exported to SPSS version 25. Results of the descriptive analysis were summarized by frequencies, means, and proportions, and presented by using tables and figures. Bivariable and multivariable logistic regressions were performed. *p* value < 0.05 was considered statistically significant.

**Result:**

A total of 315 psychiatric patients were included in the present study. The mean age (SD) of the respondents was 36.27 ± 10.85 years. ECG abnormalities were identified among 191 (60.6%) respondents. Age older than 40 years [AOR = 3.31: 95% CI 1.58–6.89], treatment with antipsychotics [AOR = 4.16: 95% CI 1.25–13.79], polytherapy [AOR = 3.13: 95% CI 1.15–8.62], having schizophrenia [AOR = 3.11: 95% CI 1.20–8.11], and illness duration of > 10 years [AOR = 4.25: 95% CI 1.72–10.49] were significantly associated with ECG abnormalities.

**Conclusions:**

In the present study, six out of ten respondents had ECG abnormalities. Age of the respondents, treatment with antipsychotics, having schizophrenia, polytherapy and illness duration of > 10 years were significant predictors of ECG abnormalities. Routine ECG investigation should be performed in the psychiatry treatment setting and further studies are recommended to delineate factors affecting ECG abnormalities.

## Background

According to the Diagnostic and Statistical Manual of Mental Disorders fifth edition (DSM-V), mental disorder is defined as a major disturbance in an individual’s thinking, feelings, or behavior that reflects a problem in mental function and causes distress or disability in social, work, or family activities [1]. World Health Organization (WHO) has reported that 10% of the world population is suffering from mental illness and one in four people meets the criteria for mental illness at some point during their life time [2].

Patients with mental disorders have two to three-fold mortality risk and 15–20-years shorter life expectancy than the general population [3]. Physical conditions account for approximately 70% of deaths in patients with mental disorders, with cardiovascular diseases (CVDs) contributing 17.4% to 22.0% to the reduction in overall life expectancy [4]. Globally, over 80% of patients with bipolar disorder have some degree of medical comorbidity with the vast majority suffering or dying from CVDs [5]. Patients with schizophrenia have been reported to be three times more likely than the general population to die from a heart attack [6]. The comorbidity of mental disorders and CVDs is of particular public health concern because CVDs are the leading cause of death globally [7]. According to the 2017 WHO’s global estimate, each year 17.8 million die from CVDs and more than 75% of these deaths occur in low and middle-income countries [8]. In 2014, approximately 30% of the Ethiopian population died as a result of noncommunicable diseases, with CVDs accounting for 9% of all deaths [9].

The high rate of CVD in psychiatric patients compromises the quality of life of both the affected and their caregivers, further affecting family finances and household productivity. Mental disorders and CVDs account for nearly 70% of global economic losses, owing to rising medical costs, increased healthcare utilization, and lost productivity [10].

Several factors contribute to the development of CVDs among psychiatric patients. Intrinsic biological changes that occur during psychosis and an increased prevalence of modifiable cardiovascular risk factors, such as obesity, smoking, diabetes, and dyslipidemia, are some of the reasons for the high rate of CVD in psychiatric patients [11]. In addition to this, treatment with psychotropic medications is also linked to the development of CVDs [12]. Such medications have been widely used to treat a wide range of mental disorders, but they are associated with a risk of various side effects including weight gain and abnormalities in glucose and lipid metabolisms which further increase the risk of CVDs [13]. Pathophysiological mechanisms such as activation of the hypothalamic–pituitary–adrenal (HPA) axis, autonomic nervous system imbalance, serotonergic dysfunction, and platelet activation explain the link between CVD and mental disorders [14].

Despite the high prevalence of CVD, about 80% of patients with mental disorders have limited access to general healthcare and fewer opportunities for CVD screening [15]. Moreover, patients with mental disorders have reduced ability to verbalize concerns, poor insight into illness, and a tendency to ignore cardiovascular symptoms such as chest pain and palpitations, which may contribute to the poor detection of CVDs [16]. Thus, underrecognition of CVDs, coupled with limited access to healthcare facilities, raises the risk of sudden cardiac death in these patients [17]. Therefore, early detection of subclinical CVDs using objective screening tools like ECG may improve patients’ clinical outcomes and reduce further disabilities and mortality associated with comorbid CVDs [18]. Some studies had previously reported the prevalence of ECG abnormalities in people with psychiatric disorders. However, most of these studies presented an incomplete picture because they focused on solitary ECG abnormalities [19–22]. Besides, the factors associated with ECG abnormalities were not well studied. On top of that, to the best of our knowledge, no study had been previously conducted on ECG abnormalities and associated factors of psychiatric patients in Jimma, in particular and in Ethiopia as a whole. Thus, this study aimed to assess ECG abnormalities and associated factors among psychiatric patients attending follow-up at Jimma Medical Center.

Early detection of ECG abnormalities may offer a simple way to identify patients with psychiatric disorders at greater cardiovascular risk so as to take preventive measures for those who are at high cardiovascular risk. The findings of this study might be helpful for policymakers and health planners for future planning to decide whether routine ECG evaluation should be indicated for specific circumstances or generally in psychiatric patients. Furthermore, this study will add additional knowledge besides the existing literature for the scientific community and it will serve as baseline data for further studies on similar area.

## Methods

### Study design and setting

An institution based cross-sectional study was conducted from October 14 to December 10, 2021 at Jimma Medical Center (JMC). JMC is a teaching and referral center which is located in Jimma town, Oromia regional state, South-west Ethiopia, at a distance of 352 km from the capital city, Addis Ababa and it provides services for approximately 15 million patients coming from south-west of Ethiopia. JMC is one of the few hospitals in Ethiopia that provides psychiatric services in both inpatient and outpatient settings. JMC Psychiatry Clinic was established in 1996. Currently, there are three Psychiatry OPD clinics and 26 beds are available for the inpatient service. On average, around 30 patients receive services at the outpatient clinic on daily basis.

### Study population and eligibility

All psychiatric patients attending follow-up at JMC Psychiatry Clinic were the source population and the study population was all selected psychiatric patients attending follow-up at JMC Psychiatry Clinic and who fulfilled the inclusion criteria. Patients with a clinical diagnosis of psychiatric disorders and who were taking follow-up treatment during the study period were included in the study. However, psychiatric patients with preexisting cardiovascular diseases, aggressive and acutely disturbed patients, pregnant women, those with serious medical conditions, and those who couldn’t give informed consent or who had no care giver to provide informed consent were excluded from the study.

### Sample size determination and sampling procedure

The sample size was determined using Epi info STAT CALC version 7.2.4.0 by considering the following assumption: prevalence of ECG abnormality among psychiatric patients 67.5% taken from a previous study done in Johannesburg [23], 95% confidence level, 5% margin error of error, and population size of 2380. Finally, a sample size of 325 was obtained by considering a 10% non-response rate.

All psychiatric patients who fulfilled the inclusion criteria were included using consecutive sampling technique until the required sample size was achieved.

### Data collection tools and procedures

Data were collected by trained BSc Nurses and Psychiatry Nurse who were employed from JMC Psychiatry Clinic. The data collectors were trained for two days prior to the data collection regarding the purpose of the study, interview, measurement techniques, and ethical issues.

An interviewer-administered structured questionnaire was used assess sociodemographic variables and behavioral factors whereas clinical characteristics and medication history were extracted from the Participants’ medical charts. The questionnaire was adapted from the WHO Stepwise approach to chronic disease risk factor surveillance. It was initially prepared in English and was translated to Amharic and Afan Oromo and retranslated back to the English language for its consistency. On each data collection day, all the collected data were reviewed by the principal investigator for completeness, accuracy, and clarity.

The WHO standard total physical activity calculation guide [24] was used to assess the physical activity status and the respondents were accordingly categorized as physically active or inactive. Psychotropic medication doses were converted into multiples of the Defined Daily Dose (DDD) by dividing the prescribed daily dose (PDD) by the DDD. The DDD of the psychotropic medications was obtained from the WHO’s Collaborative Center for Drug Statistics [25], whereas the PDD of the medications was obtained from the patients’ medical charts. For patients who were taking more than one psychotropic medication, the multiples of DDD for each drug were summed up to give a cumulative dose.

Weight and height were measured using a combined height and weight scale (made in India, manufactured date-03/2017). Weight was measured to the nearest 0.1 kg with the participant not wearing shoes and heavy clothes, whereas height was measured to the nearest 0.1 cm with the participant standing upright with the heel, buttock, and upper back along the same vertical plane, arms at the side and looking straight forward. Body mass index (BMI) was computed by dividing the weight by the square of height. Waist circumference (WC) was measured in cm using a stretch-resistant tape meter in the horizontal plane midway between the inferior margin of the ribs and the superior border of the iliac crest at the end of expiration. Blood pressure was measured on the left arm in mmHg using a calibrated sphygmomanometer (Yton sphygmomanometer, Italy, model-10220060) whilst the participant was in a sitting position, with his/her back supported, legs un crossed, arm supported, and cubital fossa at heart level after 5 min of rest. A resting 12 lead ECG calibrated at a paper speed of 25 mm/s and amplitude of 10 mm/mv was obtained in a supine position using ‘York’ ECG machine (model-YSIPL-155). Patient preparation and lead attachments were made according to the standard manual for Minnesota code protocol [26]. The ECG findings were then coded and summarized according to the Minnesota code manual of electrocardiographic findings and the electrocardiograms were interpreted by a cardiologist.

### Study variables

The dependent variable was ECG abnormality and the independent variables were Socio-demographic factors (age, sex, occupation, and place of residence), behavioral factors (smoking, khat chewing, alcohol consumption, and physical activity), disease-related factors (type of psychiatric disorder and duration of illness), medication-related factors (number of psychotropic medications, type of medication, dosage of medication and treatment duration), body composition and blood pressure.

### Operational definitions

*ECG abnormality* refers to any change deviated from normal sinus ECG based on Minnesota ECG coding criteria.

*Normal sinus rhythm* having a regular heart rate between 50 and 100 beats per minute with normal P wave, PR interval, QRS complex and T wave.

*Emotional disorders* include major depressive disorder, bipolar disorder, anxiety disorder, post-traumatic stress disorder, dysthymia, and schizoaffective disorder.

*Low dose medication* if the ratio of PDD to DDD is less than one.

*High dose medication* if the ratio of PDD to DDD is greater than one.

*Elevated blood pressure* a respondent who had a systolic blood pressure of ≥ 140 mmHg and/ a diastolic blood pressure of ≥ 90 mmHg.

### Data processing and analysis

Data were entered into Epi data version 4.6 statistical software and exported to Statistical Package for Social Science (SPSS) version 25. Data cleaning and editing were done before the actual data analysis. Descriptive statistics for frequencies, mean and standard deviation were performed to summarize the dependent and independent variables. Bivariable and multivariable logistic regressions were performed to determine the association between dependent and independent variables. Firstly, each independent variable was entered into bivariable analysis one by one. Then, variables with *p* value of less than 0.25 on bivariable analysis were entered to multiple logistic regression altogether to control confounders. Finally, variables with *p* value of ≤ 0.05 on multivariable regression were considered as predictors of ECG abnormalities. Model fitness was tested with Hosmer–Lemeshow test. Odds ratio with a 95% confidence interval was used to show the degree of association between dependent and independent variables.

## Results

A total of 325 participants were interviewed and underwent ECG examination**.** Ten of the 325 respondents were excluded from the analysis, mainly because of ECGs of poor quality and artifacts. The remaining 315 respondents were included in the analysis. The mean age of the respondents was 36.27 ± 10.85 years with a minimum of 18 and a maximum of 65 years and 187 (59.4%) respondents were men. One-fourth (25.1%) were farmers, 194 (61.6%) were rural dwellers and 121 (38.4%) respondents had attended primary education as shown in Table 1.

**Table 1:** Socio-demographic characteristics of psychiatric patients attending follow-up at JMC Psychiatry Clinic from October 14 to December 10, 2021 (n = 315)

Variables	Category	Frequency	Percentage
Sex	Male	187	59.4
	Female	128	40.6
Age (years)	18–30	119	37.8
	31–45	124	39.4
	46–59	67	21.2
	≥ 60	5	1.6
Educational status	No formal education	77	24.4
	Primary education	121	38.4
	Secondary education	72	22.9
	tertiary education	45	14.3
Occupation	Government employee	49	15.5
	Non-governmental employee	61	19.4
	Farmer	79	25.1
	Housewife	63	20.0
	Merchant	48	15.2
	Others*	15	4.8
Place of residence	Urban	121	38.4
	Rural	194	61.6
Total		315	100

*Daily laborer, student, and unemployed

### Behavioral characteristics of the respondents

Two hundred twenty-eight respondents (72.4%) respondents were non-alcohol users whereas 217 (68.9%) had never smoked tobacco products. Khat chewers accounted for 131 (41.6%). Regarding their physical activity status, 187 (59.4%) respondents reported to perform a moderate physical activity of less than 150 min or a vigorous physical activity of 75 min per week.

### Clinical characteristics of the respondents

**Fig. 1:**
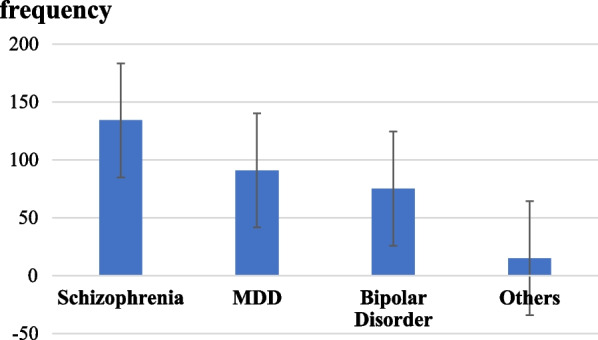
A graph showing the diagnostic category of psychiatric patients attending follow-up at JMC Psychiatry Clinic from October 14 to December 10, 2021 (n = 315)

Out of the total respondents, 134 (42.5%) were diagnosed with schizophrenia, 91 (28.9%) were diagnosed with Major Depressive Disorder (MDD), and 75 (23.8%) had bipolar disorder. Other psychiatric disorders like anxiety disorder (6, 1.9%), post-traumatic stress disorder (6, 1.9%), dysthymia (2, 0.6%) and schizoaffective disorders (1, 0.3%) totally accounted for 15 (4.8%) as shown in Fig. 1. The mean duration of illness was 7.16 ± 5.49 years with a minimum of 6 months duration and a maximum of 25 years.

### Medication related factors

The most frequently prescribed class of psychotropic medications was antipsychotic (197, 62.5%). Antidepressants were prescribed for 110 (34.9%) and mood stabilizer was prescribed for 70 (22.2%) respondents. Sodium valproate was the most frequently prescribed monotherapy (67, 21.3%) whereas the combination of risperidone and amitriptyline was the most common combination therapy (50, 15.8%). According to the classification made using the multiples of DDD of the drugs, 270 (85.7%) respondents were treated with low-dose psychotropic medications (Table 2). The mean treatment duration was 6.18 ± 4.89 years while ranging from 2 months to 24 years.

**Table 2:** Medication-related factors of psychiatric patients attending follow-up at JMC Psychiatry Clinic from October 14 to December 10, 2021 (n = 315)

Variable	Category	Frequency	Percentage
Monotherapy with psychotropic drug	Antipsychotics only	114	36.2
	Risperidone	56	17.8
	Chlorpromazine	18	5.7
	Haloperidol	40	12.7
	Antidepressants only	51	16.2
	Amitriptyline	35	11.1
	Fluoxetine	16	5.1
	Mood stabilizer only	67	21.3
	Sodium valproate	67	21.3
	Total	232	73.7
Polytherapy with psychotropic drugs	Antipsychotics with antidepressants	59	18.7
	Risperidone with amitriptyline	50	15.8
	Chlorpromazine with amitriptyline	9	2.9
	Antipsychotics with a mood stabilizer	3	1.0
	Risperidone with sodium valproate	3	1.0
	Two antipsychotics together	21	6.7
	Risperidone with chlorpromazine	21	6.7
	Total	83	26.3
Dosage of medication	Low dose	270	85.7
	High dose	45	14.3

### Anthropometric and blood pressure measurements

The mean weight of the respondents was 66.55 ± 7.67 kg. Most (239, 75.8%) of the respondents had a BMI in the range between 18.5 and 24.9 kg/m^2^ and the mean BMI was 23.19 ± 2.64 kg/m^2^. As per the WHO’s classification, 245 (77.8%) respondents had normal waist circumference whereas 19 (6.0%) respondents met the criteria for substantially increased risk waist circumference. Regarding their blood pressure measurement, 76 (24.1%) respondents had elevated blood pressure. The mean systolic and diastolic blood pressure was 118.01 ± 12.83 mmHg and 79.27 ± 8.08 mmHg respectively.

### ECG status of the respondents

**Fig. 2:**
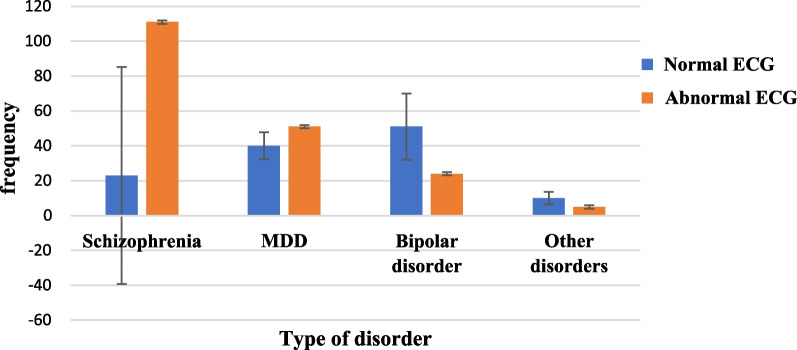
A graph showing ECG status of psychiatric patients attending follow-up at JMC Psychiatry Clinic from October 14 to December 10, 2021 (n = 315)

Out of the total 315 participants, 191 (60.6%) had at least one ECG abnormality. ECG abnormalities were 35.2% among patients with schizophrenia, 16.2% among MDD, 7.6% among bipolar disorder and 1.6% among patients with other psychiatric disorders (Fig. 2).

**Fig. 3:**
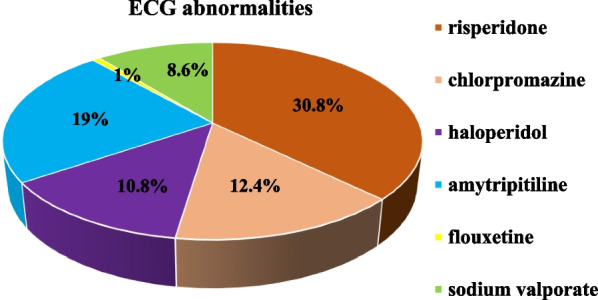
A pie chart showing the proportion of ECG abnormalities across different psychotropic medications prescribed to psychiatric patients attending follow-up at JMC Psychiatry Clinic, from October 14 to December 10, 2021 (n = 315)

As per the medications prescribed, ECG abnormalities were 48.3% among patients treated with antipsychotic medication, 20.0% among antidepressants, and 8.6% among those treated with a mood stabilizer. Ninety-seven (30.8%) respondents who were taking risperidone had ECG abnormalities. Similarly, ECG abnormalities were 19% among respondents treated with amitriptyline and 12.4% among those treated with chlorpromazine (Fig. 3).

### Types of ECG abnormalities

From the different categories of ECG abnormalities identified according to the Minnesota code, arrhythmia was the most frequently observed abnormality (87, 27.6%). QTc prolongation was identified in 61 (19.4%) and conduction block in 58 (18.4%) respondents. Fifty-three (16.8%) respondents had ST-segment abnormalities, 37 (11.7%) had axis deviation and 31 (9.8%) had chamber enlargement and hypertrophy. T wave abnormalities and Q wave abnormality were observed in 24 (7.6%) and 18 (5.7%) respondents respectively (Table 3).

**Table 3:** Types of ECG abnormalities identified among psychiatric patients attending follow-up at JMC Psychiatry Clinic from October 14 to December 10, 2021 (n = 315)

ECG abnormalities	Category	Frequency	Percentage
Arrhythmia	Sinus arrhythmia	13	4.1
	Sinus tachycardia	43	13.7
	Sinus bradycardia	19	6.0
	Atrial fibrillation	5	1.6
	Premature ventricular contraction	7	2.2
	Total	87	27.6
QTc prolongation		61	19.4
Conduction block	First degree AV block	9	2.9
	Ventricular preexcitation pattern (WPW)	15	4.8
	Incomplete LBBB	7	2.2
	Incomplete RBBB	13	4.1
	Left anterior fascicular block	9	2.8
	Left posterior fascicular block	5	1.6
	Total	58	18.4
ST-segment abnormalities	ST elevation	39	12.3
	Anterior	20	6.2
	Inferior	10	3.2
	Lateral	9	2.9
	ST depression	14	4.5
	Anterior	10	3.2
	Inferior	4	1.3
	Total	53	16.8
Axis deviation	Right axis deviation	12	3.8
	Left axis deviation	25	7.9
	Total	37	11.7
Chamber enlargement and hypertrophy	Left ventricular hypertrophy	12	3.7
	Right ventricular hypertrophy	10	3.2
	Left atrial enlargement	6	1.9
	Right atrial enlargement	3	1.0
	Total	31	9.8
T wave abnormalities	T wave flattening	20	6.3
	Inverted T wave	4	1.3
	Total	24	7.6
Q wave abnormality		18	5.7

### Factors associated with ECG abnormalities

In the bivariate analysis, variables such as age, place of residence, treatment duration, duration of illness, treatment with antipsychotics, treatment with mood stabilizer, dosage of medication, treatment type, type of psychiatric disorder, physical activity, cigarette smoking, khat chewing, and BMI were candidate variables for multiple logistic regression (*p* value < 0.25). Multivariate analysis was then performed to control confounders and five variables i.e. age, duration of illness, treatment with antipsychotics, type of psychiatric disorder, and treatment type showed statistically significant association with ECG abnormalities (*p* ≤ 0.05) (Table 4).

**Table 4:** Bivariable and multivariable analysis of factors associated with ECG abnormalities among psychiatric patients attending follow-up at JMC Psychiatry Clinic, from October 14 to December 10, 2021 (n = 315)

Variable	Category	ECG abnormality		COR (95% CI)	AOR (95% CI)
		No Freq (%)	Yes Freq (%)		
Age	≤ 40 years	106 (33.7)	95 (30.2)	1	
	> 40 years	18 (5.7)	96 (30.5)	5.95 [3.35–10.57]**	3.31 [1.58–6.89]*
Residence	Urban	41 (13.0)	80 (25.4)	1.46 [0.91–2.34]*	1.40 [0.75–2.57]
	Rural	83 (26.3)	111 (35.5)	1	
Cigarette smoking	Never	102 (32.4)	115 (36.5)	1	
	Current smoker	9 (2.9)	45 (14.3)	4.43 [2.07–9.52]**	1.70 [0.61–4.69]
	Former smoker	13 (4.1)	31 (9.8)	2.12 [1.05–4.26]*	1.23 [0.46–3.29]
Khat use	Never	42 (13.3)	60 (19.0)	1	
	Current user	48 (15.2)	95 (30.2)	1.38 [0.82–2.34]*	0.88 [0.42–1.85]
	Former user	34 (10.8)	36 (11.4)	0.74 [0.40–1.37]	1.05 [0.45–2.43]
Treatment with mood stabilizer	Yes	43 (13.7)	27 (8.6)	0.30 [0.18–.52]**	1.707 [0.64–4.53]
	No	81 (25.7)	164 (52.1)	1	
Treatment with antipsychotics	Yes	45 (14.3)	152 (48.3)	6.40 [3.87–10.60]**	4.16 [1.25–13.79]*
	No	79 (25.1)	39 (12.4)	1	
Type of psychiatric disorder	Schizophrenia	23 (7.3)	111 (35.2)	6.10 [3.57–10.42] **	3.11 [1.20–8.11]*
	Emotional disorders	101 (32.1)	80 (25.4)	1	
Duration of illness	≤ 10 years	107 (34.0)	108 (34.3)	1	
	> 10 years	17 (5.4)	83 (26.3)	4.84 [2.70–8.70] **	4.25 [1.72–10.49]*
Treatment duration	≤ 10 years	119 (37.8)	153 (48.6)	1	
	> 10 years	5 (1.6)	38 (12.1)	5.91 [2.26–15.48]**	0.69 [0.17–2.81]
Treatment type	Monotherapy	110 (34.9)	122 (38.7)	1	
	Polytherapy	14 (4.4)	69 (21.9)	4.44 [2.37–8.34]**	3.13 [1.15–8.62]*
BMI category	Normal	101 (33.3)	138 (43.8)	1	
	Overweight	18 (5.7)	49 (15.6)	1.99 [1.09–3.62]*	1.17 [0.54–2.54]
	Obese	5 (1.6)	4 (1.3)	0.59 [0.15–2.23]	0.27 [0.05–1.47]
Physical activity status	Inactive	62 (19.7)	126 (40.0)	1.94 [1.22–3.08]*	1.79 [0.93–3.46]
	Active	62 (19.7)	65 (20.6)	1	
Dosage of medication	High dose	12 (3.8)	33 (10.5)	1.95 [0.96–3.94]*	0.75 [0.28–2.01]
	Low dose	112 (35 (.6)	158 (50.2)	1	

*p < 0.05; **p < 0.001

The numbers written in bold show variables which have significant association with the dependent variable

Accordingly, by putting all other variables constant, the odds of ECG abnormalities were three times higher in patients older than 40 years than younger age group (AOR = 3.31: 95% C.I 1.58–6.89: *p* value = 0.002). Psychiatric patients who were treated with antipsychotics were four times more likely to have ECG abnormalities than those who were treated with other psychotropic medications (AOR = 4.16: 95% CI 1.25–13.79: *p* value = 0.019). Patients with schizophrenia had a three-fold risk of ECG abnormalities as compared to patients with emotional disorders (AOR = 3.11: 95% CI 1.20–8.11: *p* value = 0.02). Similarly, patients treated with polytherapy had a three-fold risk of ECG abnormalities as compared to those treated with monotherapy (AOR = 3.13: 95% CI 1.15–8.62: *p* value = 0.027). Patients who were ill for more than 10 years were four times more likely to have ECG abnormalities than patients with lower illness duration (AOR = 4.25: 95% CI 1.72–10.49: *p* value = 0.002).

## Discussion

People with psychiatric disorders have increased risk of cardiovascular morbidity and mortality as compared with the general population [27]. Early detection of ECG abnormalities may offer a simple way to identify psychiatric patients who are at higher risk of CVDs. With the objective of assessing ECG abnormalities and associated factors, an institution-based cross-sectional study was conducted among psychiatric patients attending follow-up at JMC. The mean age of the respondents was 36.27 ± 10.85 years and 59.4% were males.

The prevalence of ECG abnormalities among psychiatric patients was 60.6%. This finding is higher than the previous studies conducted in Denmark (28%) [16], Minnesota (54%) [28], and two studies conducted in Switzerland (17.9%, 27.3%) [19, 29]. The present finding is however lower than the study done in Johannesburg (67.5%) [23]. These inconsistencies in the prevalence rate of ECG abnormalities could be attributed to the differences in socioeconomic status, sample size, study design, inclusion criteria, drug prescribing pattern, and criteria for ECG abnormality.

According to the present study, arrhythmia was the most common ECG abnormality which was observed in 27.6% of the respondents. In contrast to the present finding, a lower prevalence of arrhythmia was reported in the studies conducted in Minnesota (20%) [28] and Switzerland (6.3%) [29]. The lower percentage reported in Minnesota might be due to the small sample size involved in the study (37 respondents). By the same token, the study done in Switzerland defined sinus tachycardia at a cut value of > 120 beats per minute which probably contributed to the lower rate of arrhythmia reported in the study. The possible explanation for the occurrence of arrhythmia may be the fact that psychiatric patients have episodes of highly sympathomimetic arousal during restraint, which may trigger potentially fatal arrhythmias. In addition, psychotropic medications also have an arrhythmogenic effect through blockage of peripheral cholinergic and adrenergic receptors [30, 31].

The current study revealed that 18.4% of the respondents had conduction block. This finding is in accordance with the study done in Denmark (14.5%) [16]. Blockade of high-voltage L-type calcium channels that are responsible for conduction via the atrioventricular node may explain the mechanism of conduction abnormalities in psychiatric patients. Psychotropic medications, specifically antipsychotics and TCAs, cause sodium ion channel blockage. This reduces the inward Na^+^ depolarizing current leading to conduction delay, atrioventricular block, and bundle-branch block [32].

In the present study, ST-segment abnormalities were identified in 16.8% of the respondents. On the other side, the study done in Nigeria [33] reported 5% prevalence of ST-segment abnormalities. Treatment with psychotropic medications has been associated with blockade of the fast sodium current encoded by the sodium channel, voltage-gated type V α-subunit. This blockade reduces peak sodium influx causing altered voltage gradients which manifest as ST-segment elevation on the ECG [34].

According to the present study, 9.8% of the participants were found to have chamber enlargement or hypertrophy. This report is consistent with the study done in Denmark [16] which reported an 8.5% prevalence of chamber enlargement among the respondents. Autonomic nervous system imbalance characterized by sympathetic hyperactivity might explain the mechanism of cardiac hypertrophy in these patients [35].

In the current study, T wave abnormalities were found in 7.6% of the respondents. The present finding is lower than the finding of the study in Johannesburg (15.4%) [23]. As to why our finding is lower, the study in Johannesburg [23] reported treatment with additional psychotropic drugs like carbamazepine which is not reported in the present study but is known to be related to T wave abnormalities [36]. Sympathetic overactivity and the effect of antipsychotics on serum potassium level may explain the T wave abnormality that occur in psychiatric patients. Antipsychotics are believed to cause hypokalemia by changes in adrenergic activity. It was postulated that a hyperadrenergic state might drive beta-2-receptor stimulation, causing an influx of potassium into skeletal muscle, resulting in a hypokalemic trend [37].

According to the present finding, age older than 40 years was one of the predictors of ECG abnormalities. This finding is supported by the previous studies conducted in Atlanta [21], Nigeria [33], Minnesota [28], and Switzerland [29] which showed significant association between old age and ECG abnormalities. Ageing produces structural and functional changes in the cardiovascular system, which involves the appearance of cardiovascular diseases. The consequences of these conditions are the appearance of vascular stiffness, fibrosis, hypertrophy, and the involution of muscular tissue, valves, and arteries [38]. In healthy population studies, Van der Ende and coll previously observed that increasing age was associated with a linear increase of PR and QT intervals and with a weak increase of P and QRS waves duration [39].

In the current study having schizophrenia was significantly associated with ECG abnormalities. In agreement with this finding, the study done in Denmark [16] reported that schizophrenia patients had a high rate of ECG abnormality in comparison with other psychiatric disorders. The possible explanation is that schizophrenia is associated with physiologic changes, including sympathetic nervous system activation, cardiac rhythm disturbances, systemic and localized inflammation, and hypercoagulability that negatively influence the cardiovascular system [40]. Genetic studies suggest that the pathobiology of schizophrenia involves various voltage-gated ion channels. Because these proteins also control cardiac electrophysiology, variants in their encoding genes (*KCNH2*, *CACNA1C*) may increase arrhythmia and SCD risk [41, 42]. The cardiac adverse effects of the medications used to treat this disorder might be another possible explanation for the high rate of ECG abnormalities in these patients [4].

In this study, treatment with antipsychotic medication has shown a significant association with ECG abnormalities. In accordance with this finding, previous studies done in Switzerland [19], Italy [43], and Atlanta [21] showed a significant association between ECG abnormalities and treatment with antipsychotics. This could be due to the effect of antipsychotics on cardiac ion channels [44]. Antipsychotic drugs have been reported to block ion channels in the ventricles. For example, some antipsychotic medications (e.g., chlorpromazine, clozapine, and thioridazine) suppress the rapidly activating delayed rectifier current (I_Kr)_, and the QT prolongation most commonly occurs secondary to the I_Kr_ block [45]. In addition, antipsychotics are known to cause autonomic nervous system dysfunction through blockade of peripheral dopamine receptors, thus increasing sympathetic activity. Some antipsychotic medications, particularly second-generation antipsychotics, cause immunoglobin E-mediated hypersensitivity reaction, hyper-eosinophilic syndrome with direct cardiotoxic effects of eosinophils. This in turn leads to the development of drug-induced cardiomyopathy [46].

According to this study, taking polytherapy was a significant predictor of ECG abnormalities. This is consistent with two studies done in Italy [43, 47] which revealed a significant association between ECG abnormalities and polytherapy. The possible explanation could be that many psychotropic drugs share the capacity to inhibit cardiac ion channels, particularly, HERG potassium channels, therefore those agents may have synergic effects when used in combination [48]. Furthermore, several psychotropic drugs block in vitro calcium channels of the L-type. In contrast to low-voltage calcium ion channels (T-type) located in pacemaker cells, high voltage channels of the L-type modulate conduction through the sinoatrial pathway and the atrioventricular node. This mechanism may explain the unusual occurrence of second-degree sinoauricular (Mobitz type II) or atrioventricular block [49].

In the current study, psychiatric illness duration of more than 10 years was significantly associated with ECG abnormalities. This finding is in agreement with the study in Norway [50] which revealed a high cardiometabolic risk among psychiatric patients with long duration of illness. The possible explanation for this might be the long-term effects of biological changes caused by psychiatric illness. In addition, the long duration of psychiatric illness is a proxy for the duration of exposure to psychotropic medications. The long-term exposure to antipsychotic drugs places a patient with a longer illness duration at a greater risk of cardiometabolic disorders [50, 51].

### Limitation of the study

Although the present study provides helpful information in the scarce data situation of Ethiopia, it has some flaws that should be addressed in future researches. One of the study's potential drawbacks was the cross-sectional nature of the study which is not suitable to establish a causal inference. Additionally, due to financial constraints, biochemical measures such as lipid profile and serum electrolytes were not performed.

## Conclusion

In the present study, six out of ten respondents had ECG abnormalities. Age older than 40 years, treatment with antipsychotics, having schizophrenia, polytherapy and longer duration of psychiatric illness (> 10 years) were significant predictors of ECG abnormalities. Therefore, an integrated care that involves a multi-professional approach, paying attention to the early detection of coexisting cardiac disease and routine ECG investigation should be performed in the psychiatry treatment setting. Further researches with better study designs and large-scale studies are recommended to delineate factors affecting ECG abnormalities.

## Data Availability

The data sets used and/or analyzed during this study are available from the corresponding author on reasonable request.

## Abbreviations

AOR: Adjusted odds ratio
BMI: Body mass index
CI: Confidence interval
COR: Crude odds ratio
CVD: Cardiovascular disease
DDD: Defined daily dose
ECG: Electrocardiogram
JMC: Jimma Medical Center
MDD: Major depressive disorder
PDD: Prescribed daily dose
SPSS: Statistical Package for Social Science Studies
WHO: World Health Organization
